# Supplementary material for: Cell Growth Inhibition of Saponin XII from Dipsacus japonicus Miq. on Acute Myeloid Leukemia Cells
Source: Molecules. 2020 Jul 22;25(15):3325. doi: 10.3390/molecules25153325 (PMC7435407; doi:10.3390/molecules25153325)
Supplement: Supplementary file 1 [file molecules-25-03325-s001.pdf]

Supplementary Matrial

Cell Growth Inhibition of Saponin XII from *Dipsacus japonicus* Miq. on Acute Myeloid Leukemia Cells

Ba Thi Cham [1,2], Nguyen Thi Thuy Linh [1,2], Do Thi Thao [1,3], Nguyen Thi Hoang Anh [1,2], Nguyen Thanh Tam [1,2], Bui Kim Anh [2], Isabella Muscari [4], Sabrina Adorisio [5], Tran Van Sung [2], Trinh Thi Thuy [1,2,*] and Domenico V. Delfino [5,6,*]

[1] Department of Chemistry, Graduate University of Science and Technology, Vietnam Academy of Science and Technology (VAST), 18 Hoang Quoc Viet, Nghia Do, Cau Giay, 100000 Hanoi, Vietnam; BaThiCham@ich.vn (B.T.C.); NguyenThiThuyLinh@ich.vn (N.T.T.L.); DoThiThao@ich.vn (D.T.T.); NguyenThiHoangAnh@ich.vn (N.T.H.A.); NguyenThanhTam@ich.vn (N.T.T.)

[2] Department of Natural Products Research, Institute of Chemistry, VAST, 18 Hoang Quoc Viet, Nghia Do, Cau Giay, 100000 Hanoi, Vietnam; BuiKimAnh2@ich.vn

[3] Institute of Biotechnology, VAST, 18 Hoang Quoc Viet, Nghia Do, Cau Giay, 100000 Hanoi, Vietnam

[4] Section of onco-hematology, Department of Medicine, University of Perugia, 06132 Perugia, Italy; isa.muscari2@gmail.com

[5] Foligno Nursing School, Department of Medicine, University of Perugia, 06132 Perugia, Italy; adorisiosabrina@libero.it

[6] Section of Pharmacology, Department of Medicine, University of Perugia, Piazzale Severi, S. Andrea delle Fratte, 06132 Perugia, Italy

* Correspondence: thuy@ich.vast.vn (T.T.T.); domenico.delfino@unipg.it (D.V.D.); Tel.: +39-075-585-8328 (D.V.D.)

Academic Editor: Roberto Fabiani

Received: 03 July 2020; Accepted: 19 July 2020; Published: date

List of Figures

Figure S1: ^{1}H NMR spectrum of saponin XII (500 MHz, CD_3OD)**Figure S1a:** ^{1}H NMR spectrum (region from 5.4 to 3.8 ppm) of saponin XII

Figure S1b: ^{1}H NMR spectrum (region from 2.9 to 0.6 ppm) of saponin XII

Figure S2: Analytical HPLC chromatogram of isolated saponin XII

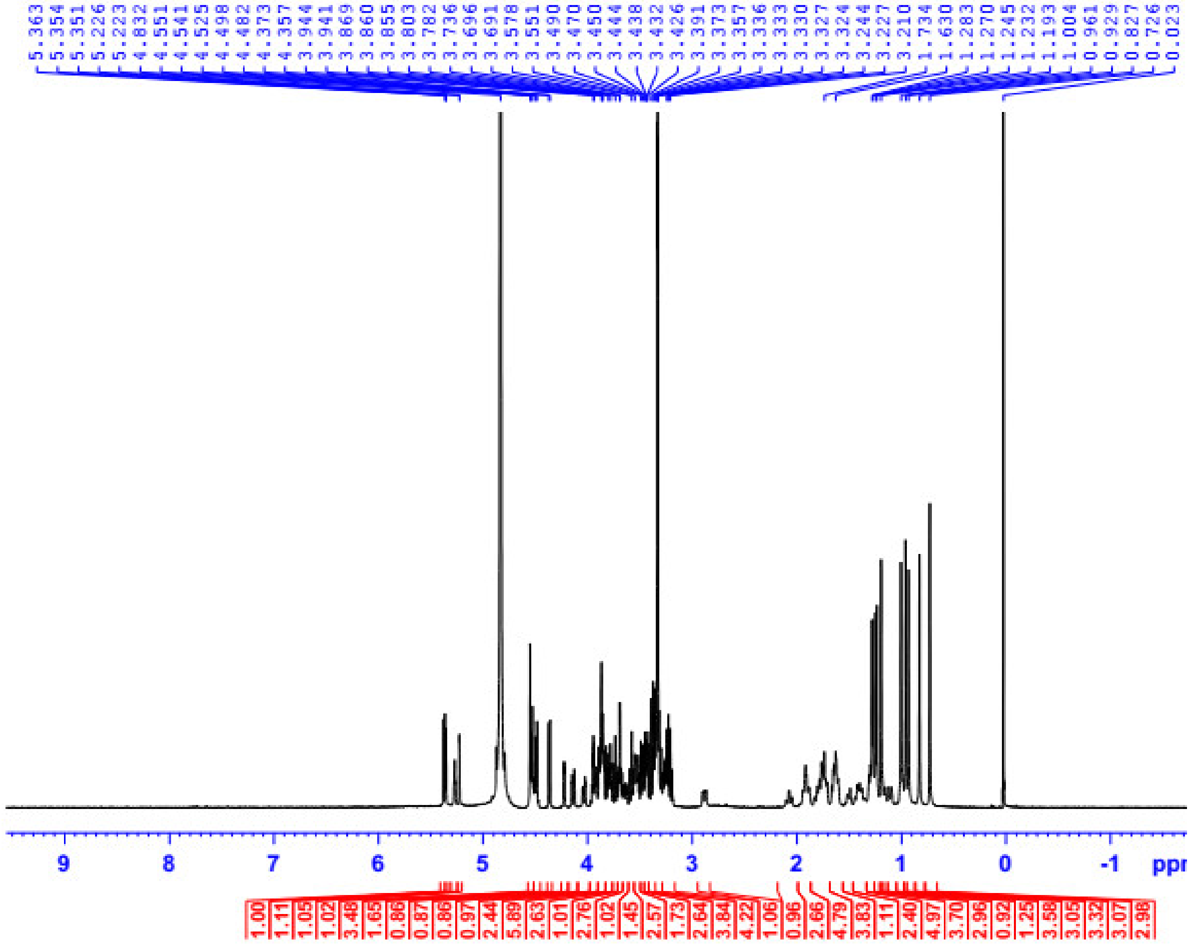

Figure S1. ^{1}H NMR spectrum of saponin XII (500 MHz, CD_3OD).

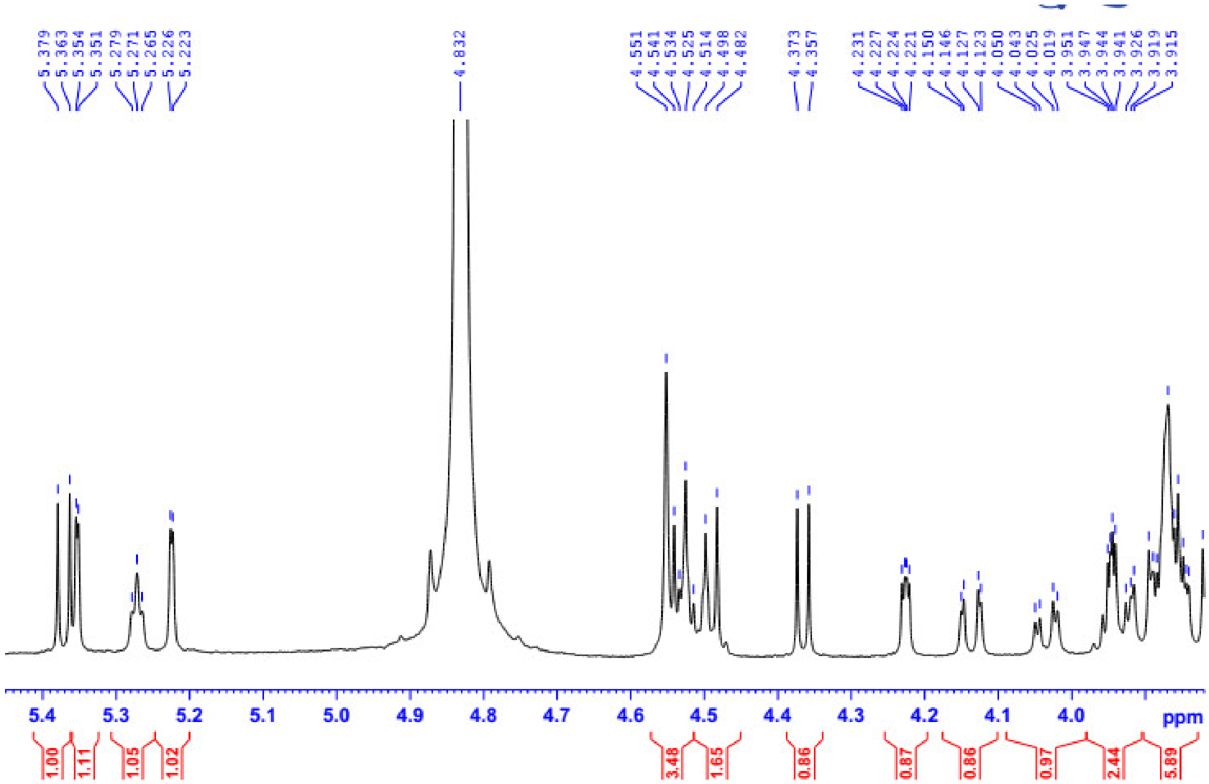

Figure S1a. ^{1}H NMR spectrum (region from 5.4 to 3.8 ppm) of saponin XII.

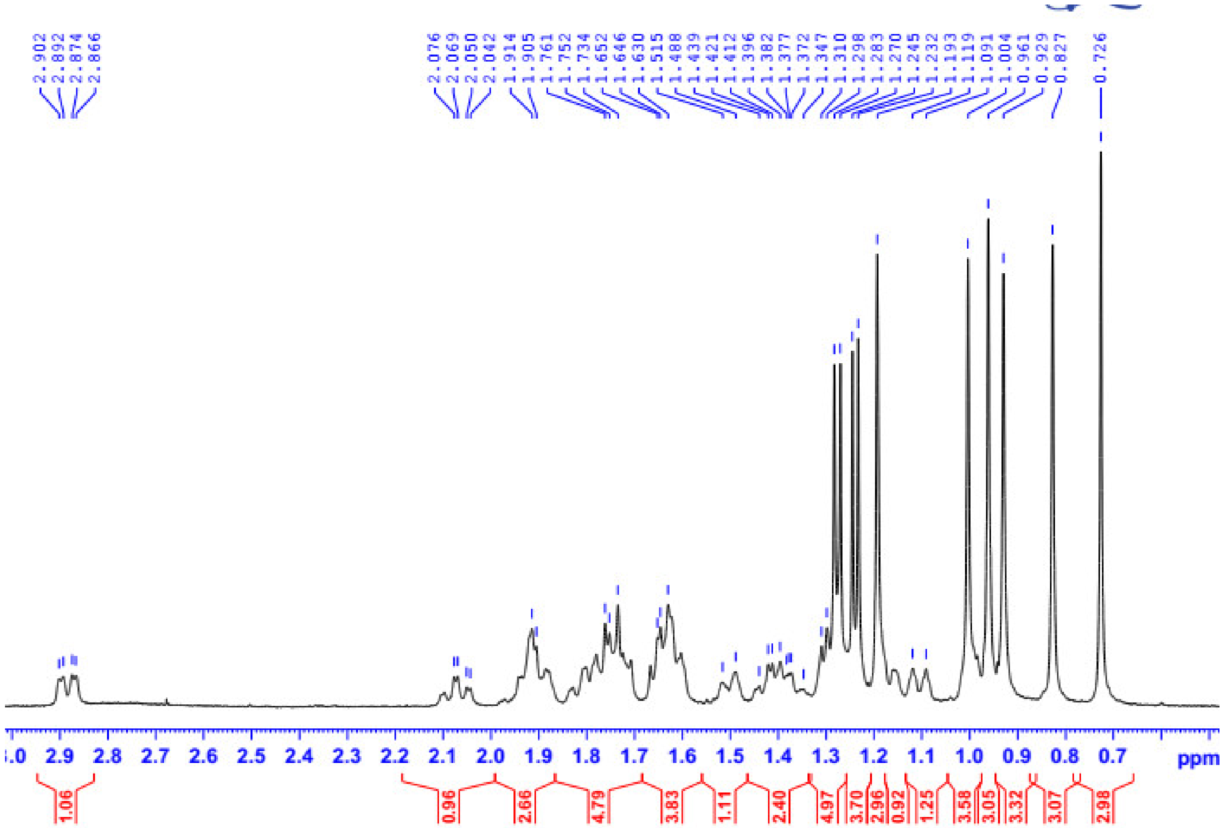

Figure S1b: ^{1}H NMR spectrum (region from 2.9 to 0.6 ppm) of saponin XII.

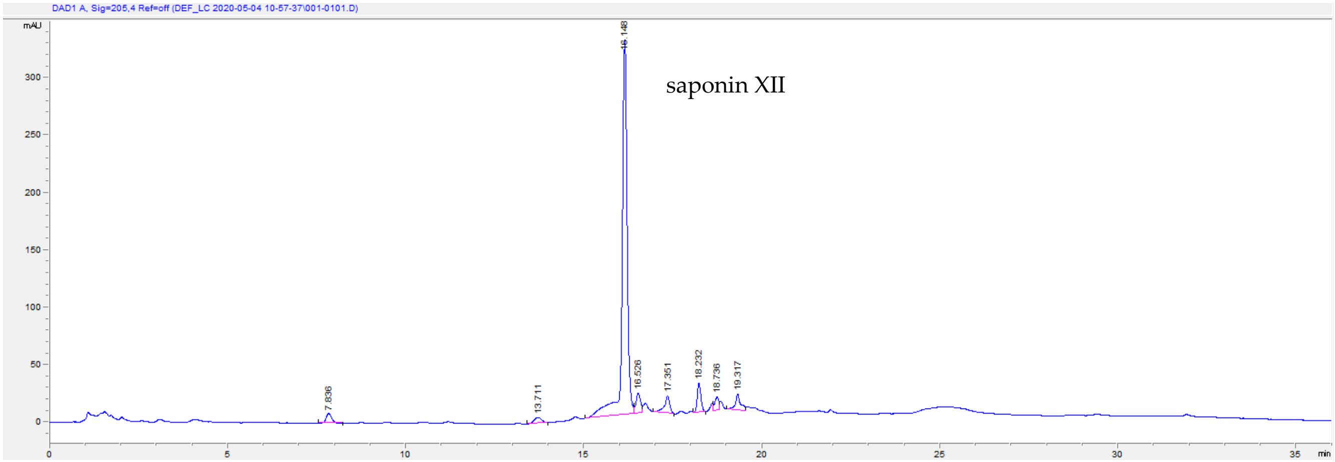

Time (min), at wavelength 205 nm

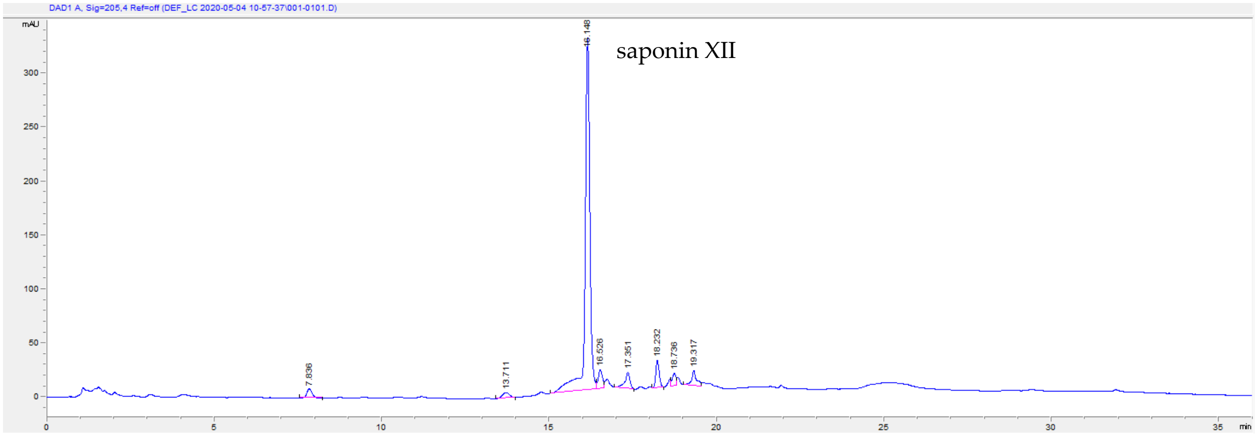

Time (min), at the wavelength 230 nm

Figure S2. Analytical HPLC chromatogram of isolated saponin XII (retention time 16.2 min).

[Column J'sphere C18 column, 150 × 4.6 mm, 5 μm, 1.0 mL/min, isocratic elution (acetonitrile in water, ACN/H_2O 20–90 %), PDA monitoring at 205 and 230 nm]
